# Characterization of a Laser Surface-Treated Martensitic Stainless Steel

**DOI:** 10.3390/ma10060595

**Published:** 2017-05-29

**Authors:** S. R. Al-Sayed, A. A. Hussein, A. A. Nofal, S. I. Hassab Elnaby, H. Elgazzar

**Affiliations:** 1 National Institute of Laser Enhanced Sciences (NILES), Cairo University, Giza 12611, Egypt; selnaby@niles.edu.eg; 2 Faculty of Engineering, Cairo University, Giza 12611, Egypt; aahussein41@yahoo.com; 3 Central Metallurgical Research and Development Institute (CMRDI), Helwan 11731, Egypt; adelnofal@hotmail.com (A.A.N.); h_elgazzar@yahoo.com (H.E.)

**Keywords:** laser surface treatment, martensitic stainless steel, macrohardeness and microhardeness, impact toughness, wear resistance, potentiodynamic polarization technique

## Abstract

Laser surface treatment was carried out on AISI 416 machinable martensitic stainless steel containing 0.225 wt.% sulfur. Nd:YAG laser with a 2.2-KW continuous wave was used. The aim was to compare the physical and chemical properties achieved by this type of selective surface treatment with those achieved by the conventional treatment. Laser power of different values (700 and 1000 W) with four corresponding different laser scanning speeds (0.5, 1, 2, and 3 m·min^−1^) was adopted to reach the optimum conditions for impact toughness, wear, and corrosion resistance for laser heat treated (LHT) samples. The 0 °C impact energy of LHT samples indicated higher values compared to the conventionally heat treated (CHT) samples. This was accompanied by the formation of a hard surface layer and a soft interior base metal. Microhardness was studied to determine the variation of hardness values with respect to the depth under the treated surface. The wear resistance at the surface was enhanced considerably. Microstructure examination was characterized using optical and scanning electron microscopes. The corrosion behavior of the LHT samples was also studied and its correlation with the microstructures was determined. The corrosion data was obtained in 3.5% NaCl solution at room temperature by means of a potentiodynamic polarization technique.

## 1. Introduction

The composition and surface microstructure of a component play a decisive role in determining its surface-dependent engineering properties (hardness, wear, fatigue, corrosion and oxidation resistance). Surface engineering processes aim to the control heating and cooling of metallic materials, to alter their physical and mechanical properties without affecting the bulk material. Conventionally, surface treatment processes like flame hardening, induction hardening, carburizing, nitriding, etc. are usually employed in enhancing the wear resistance of Fe-based alloys. These processes suffer from numerous restrictions, i.e., time and energy consumption, complex heat treatment schedule, non-controllable heat affected zones, lack of solid solubility limit, and the necessity of a quenchant [1,2,3].

On the other hand, when a high powered laser beam is used as a source of heat for surface treatment, it will obviate most of the restrictions observed in the conventional surface treatment [4,5]. Martensitic stainless steels are extensively used in engineering applications such as steam and water valves, pumps, screw machining parts, turbines, and many other applications which necessitate high strength and high resistance to wear and corrosion [6,7,8]. Failure of such stainless steel components due to corrosion, fatigue and abrasion is most likely to initiate from the surface. The engineering solution for minimizing or eradicating such surface-initiated failures lies in tailoring the surface composition and/or microstructure of the near surface region of a component without affecting the bulk [9]. In recent years, application of laser surface modification to prolong the service life of the engineering components is one of the most promising techniques for improving the tribological properties of the majority of metals. Among the different types of laser surface treatment processes, laser transformation hardening (LTH) and laser surface melting (LSM) are the simplest, as no additional materials are introduced, and they are particularly effective for processing ferrous alloys with grain refinement and uniformity of the acquired structure. In fact, these processes have been employed for improving the erosion and corrosion resistance of a number of ferrous alloys [9,10,11,12]. Only martensitic grades of stainless steel contain sufficient carbon to be laser hardened. The degree of hardening is strongly dependent on the initial condition of the steel, as well as its chromium content. Laser heating of martensitic stainless steels (MSS) in their annealed delivery condition (a microstructure of ferrite and carbides) causes a significant amount of carbide dissolution. Sufficient carbon is released to form martensite on cooling, although austenite may be retained in regions of particularly high carbon concentration [13]. Ion et al., worked on 12C27 steel. It was observed that laser surface heating resulted in redistribution of carbon to produce hardening by martensitic transformation, but the high alloy content resulted in depression of the martensite finish temperature and, consequently, the retention of austenite at room temperature [14]. Tsay et al. examined the impact toughness of AISI 403 MSS plate and laser-hardened specimens tempered at various temperatures [15]. Tianmin et al. [16] studied the impact of wear behavior of hypo-eutectoid 2Cr13 MSS, and the results of scanning electron microscopy (SEM) analysis and surface profile measurement suggested that the size of the impact wear scars of 2Cr13 specimens decreased with an increase in surface hardness. The effect of processing conditions on the corrosion performance of laser surface-melted of MSS was studied as well, on different grades of MSS [11,17,18,19,20]. The corrosion resistance of all laser surface-melted specimens was significantly improved, as evidenced by a shift from active corrosion to passivity. Steel AISI 416 is “a free machining type of martensitic stainless steel grade, with a minimum sulfur content of 0.15%”. Its practical potential is noteworthy and is frequently used in automatic screw-machined components, gears, bolts, valves, pump shafts and turbine blades. The aim of this work is to gain improvement in steel wear resistance and impact toughness as well via LHT, particularly in light of the rather scarce studies reported in this respect [21]. 

## 2. Materials and Methods 

### 2.1. Materials and Laser Treatment

The martensite steel AISI 416 used in this work has the principal alloying elements (in wt. %) as follows 0.167% C; 12%·Cr; 0.475% Mn; 0.225% S; 0.0232% P; 0.27% Ni; 0.131% Cu, and 0.194% Si. It was received in an annealed condition with a hardness of 155 Hv, in the form of round disks of diameter 150 mm and a thickness of 7.5 mm. Samples measuring 55 mm×10 mm×7.5 mm in size were machined. Preceding the laser treatment, all necessary precautions and safety measures were taken [22]. Precautions with clamping the specimen to circumvent any misalignment with the laser beam scanning were taken as well [23]. 

A Rofin sinar 2.2 KW continuous wave diode pumped Nd:YAG laser was used to generate a beam which was delivered to the rotating head by a fiber optic. The rotating head moves with variable speeds up to 9 m·min^−1^. The oscillation mode is a multimode with Gaussian energy distribution because the YAG laser has high amplification gain and a refractive index that tends to be non-uniform due to the temperature gradient inside the rod. The laser radiation focused on the sample surface by means of optics consisted of a focusing lens of focal length f = 120 mm, producing a beam spot size of 2 mm on the substrate. The laser treatment was carried out in argon shielding gas with a flux of 10 L·min^−1^ to minimize the oxidation of the hardened surface. Prior to the LHT, the surface of the AISI 416 martensitic stainless steel samples was cleaned with acetone. Preliminary attempts with different values of laser power (P) were carried out, for example 2000 and 1800 W. Unfortunately these trials resulted in the melting of the material surface and changing its dimensions. Therefore, we reduced the range of laser power to the following values to be sure that we are in the hardening range. This range of laser scanning speeds with the laser power was chosen to determine the optimum processing conditions for surface treatment. In the present investigation, two different laser powers were attempted, with four different laser scanning speeds (as listed in Table 1, with the corresponding samples are labeled as LHT P-X-Y, where X: laser power, Y: scanning speed) in order to attain different hardened layer depths. Laser surfacing was achieved by parallel tracks with 25% overlap. Since the laser spot has 2 mm diameter, and in order to achieve a smooth and homogenous track profile, we had to move the laser beam between 2 mm and 1 mm (0% to 50% overlap). We chose to make a 25% overlap to avoid any overheating and any discontinuation in the profile.

For comparison, conventional heat-treated samples (CHT) were subjected to the following heat treatment; samples were austenitized at 980 °C for 15 min with a heating rate of 250 °C/h, and subsequently oil quenched.

### 2.2. Metallographic and Microstructural Analysis

Metallographic samples were prepared using standard procedures of mechanical polishing, and then were etched with acidic ferric chloride solution (25 g of FeCl_3_, 25 mL of HCl and 100 mL of H_2_O) for 10 s. The microstructures of the samples were characterized using the Axiotech 30 optical microscope (Lukas Microscope Service, Inc., Hillview Court, Mundelein, IL, USA), and the hardened layer was examined by the back-scattered electron mode of the QUANTA FEG 250 SEM (FEI, Hillsboro, OR, USA) attached with an energy dispersive X-ray (EDX) microanalyzer. The phases present in the surface layer were determined by X-ray diffraction (XRD) using CuKα radiation.

Vickers hardness values of the as-received (AR), CHT and LHT samples were measured under a load of 30 kg. The measured hardness values were an average of five readings for each condition. Additionally, the distribution of Vickers microhardness along the hardened depth to the base metal (BM) was measured under a load of 200 g for 20 s.

### 2.3. Tribology Test

The macroscopic wear performances of CHT and LHT samples were evaluated at room temperature by means of a pin-on-ring type tribometer (TNO, The Hague, Netherlands), according to ASM G 132 [24]. In this case, the ring (diameters of 73 mm) of stainless steel with hardness of 63 HRC is rotated on the stationary wear sample, which functions as the pin. The dimensions of the specimens were 7 mm × 7.5 mm × 10 mm. Before each test, the ring was rotated to a defined starting point. The experimental conditions without lubricant used for the wear tests were; normal loads =50 N (0.3 bar) and fixed sliding at speeds =8.4 m·min^−1^ (100 rpm). The wear rate was measured by a weighing method, and each specimen was weighed before and after wear testing by using a sensitive digital balance of accuracy 10^−4^ g. The measured weight loss was an average of three tested samples for each condition.

### 2.4. Impact Test

The impact test was performed at 0 °C by a Model JB-W500 impact tester (TIME Group Inc., Beijing, China). The impact machine had a maximum impact energy of 500 J, a maximum impact speed of 5.4 m·s^−1^, a minimum impact energy resolution of 0.2 J, and a rising angle of 150°. The test ascertained whether the material was tough or brittle. Un-notched samples measuring 7 mm × 10 mm × 55 mm were used [15]. The result is usually reported as the energy in J required to fracture the test piece. To carry out the test, the standard specimen is supported at its two ends on an anvil, and is struck on the opposite face to the laser treated surface, by the pendulum.

### 2.5. Potentiodynamic Corrosion Test

The corrosion resistance of the AR, CHT and LHT samples was assessed by means of electrochemical measurements. Linear polarization measurements were performed using an Autolab PGSTAT 302N potentiostat system, which was driven by NOVA 1.10 software (Eco-chemie, Utrecht, Netherlands). The measurements were achieved using a conventional three-electrode setup, where a test sample with approximately 0.2 cm^2^ exposed surface area was placed in 3.5% NaCl (pH 7) solution at 23 °C. A constant potential scan rate of 1 mV·s^−1^ was used. All potentials were computed with respect to Ag/AgCl, with a 3 mol/L KCl electrode as a reference electrode. A platinum electrode was used as the counter electrode for current measurement.

## 3. Results

### 3.1. Microstructural Analysis

#### 3.1.1. Microstructure of the As-Received Specimen 

**Figure 1:**
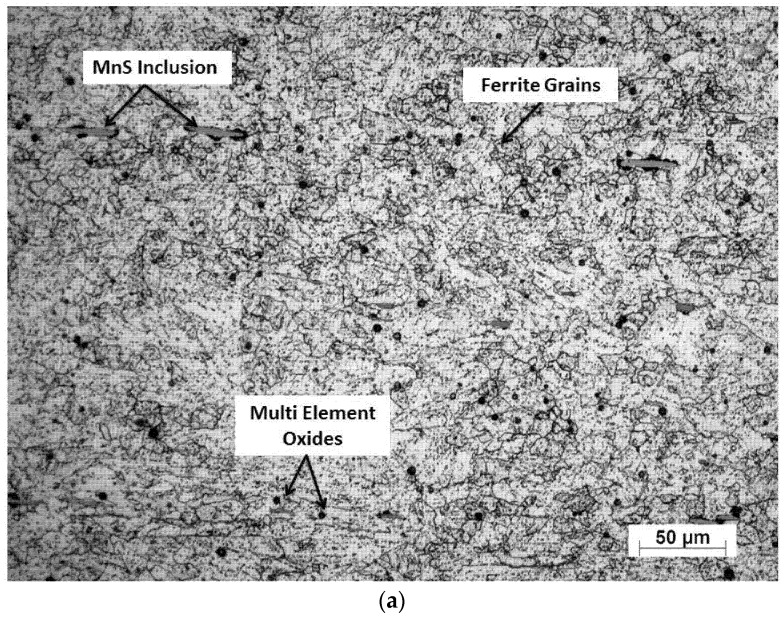

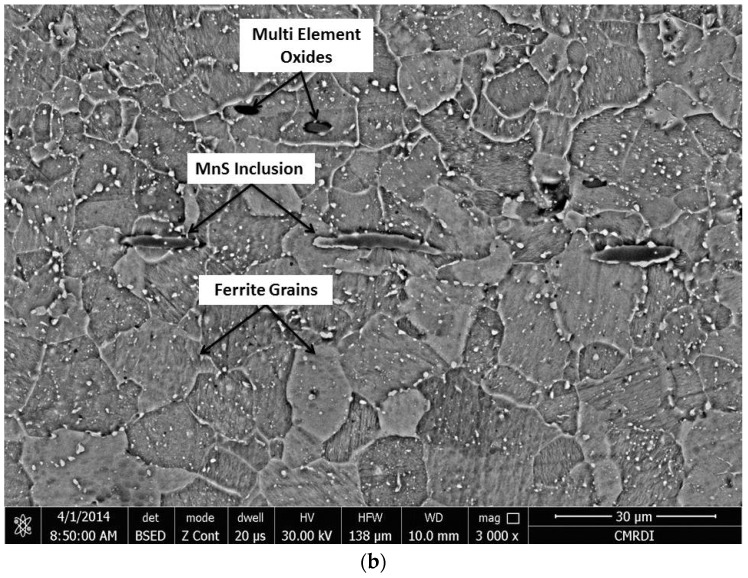
(**a**) Optical image of microstructure (magnification 200×) and (**b**) scanning electron microscopy (SEM) image of microstructure (magnification 3000×) of the as-received (AR) sample.

**Figure 2:**
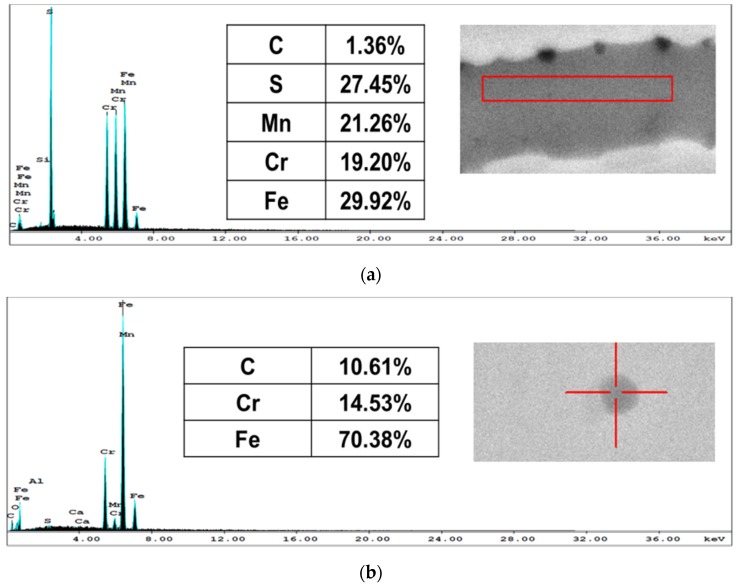

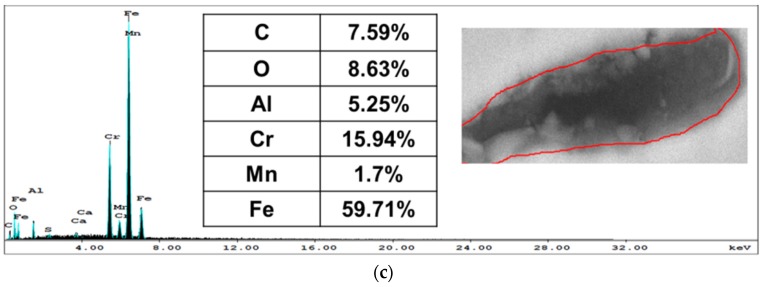
Energy dispersive X-ray (EDX) analyses of AR AISI416: (**a**) MnS inclusions; (**b**) carbide particles; and (**c**) multi-element oxides.

The microstructure of the annealed AR AISI 416 steel consisted of equiaxed or near equiaxed ferrite grains as shown in Figure 1, with MnS inclusions, carbide particles, and multi-element oxides. These constituents were identified by EDX (Figure 2). 

#### 3.1.2. Microstructure of the CHT Specimens

**Figure 3:**
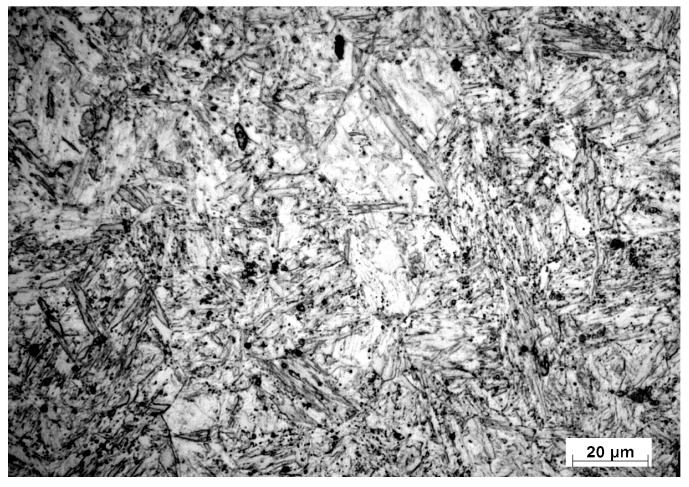
Optical image of microstructure (magnification 200×) of conventionally hardened AISI 416 stainless steel.

**Figure 4:**
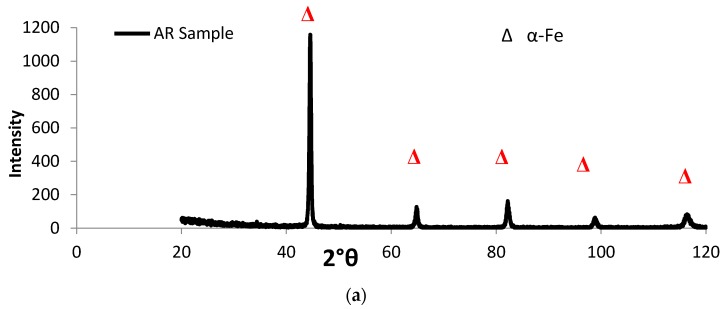

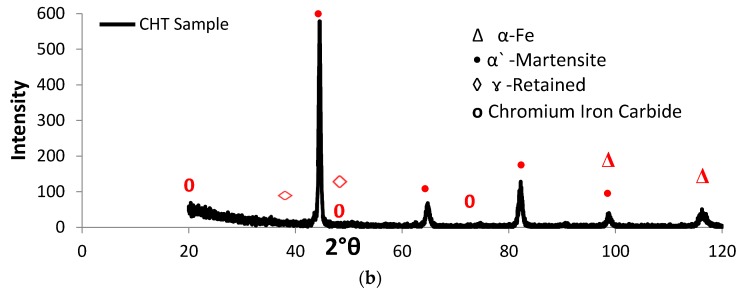
X-ray diffraction pattern of (**a**) untreated AISI 416 and (**b**) conventionally heat treated (CHT) samples.

Martensitic structures appeared in the CHT specimens (oil quenched from austenite), as shown in Figure 3. Carbide particles, multi-element oxides and MnS inclusions were observed as well. In addition, these phases were present in both AR and CHT specimens as detected by the XRD spectra shown in Figure 4.

#### 3.1.3. Microstructure of the LHT Specimens

**Figure 5:**
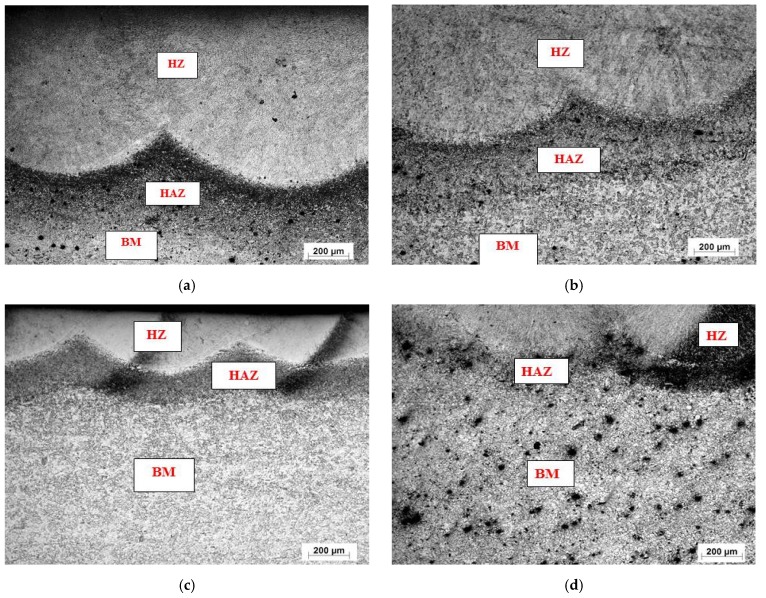
Typical microstructure features (magnification 50×) of laser heat treated (LHT) AISI 416 stainless steel at a constant power of 1000 W and different laser scanning speeds: (**a**) 0.5; (**b**) 1; (**c**) 2; and (**d**) 3 m·min^−1^.

**Figure 6:**
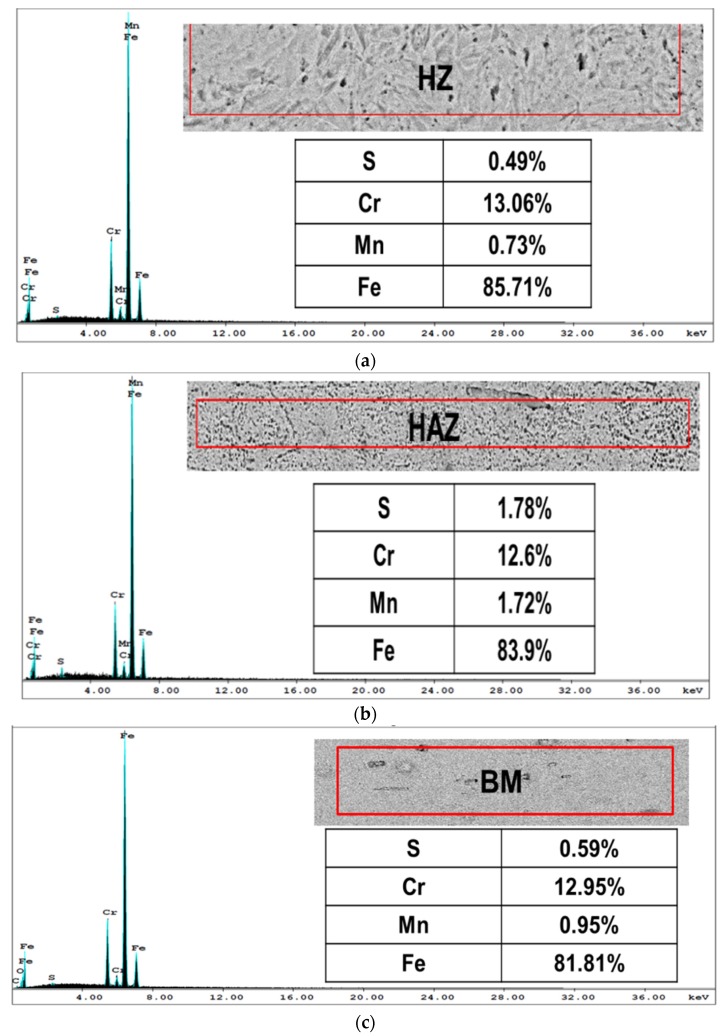
EDX analysis of the LHT P-1-0.5 sample: (**a**) hardened zone (HZ); (**b**) heat affected zone (HAZ); and (**c**) base metal (BM).

**Figure 7:**
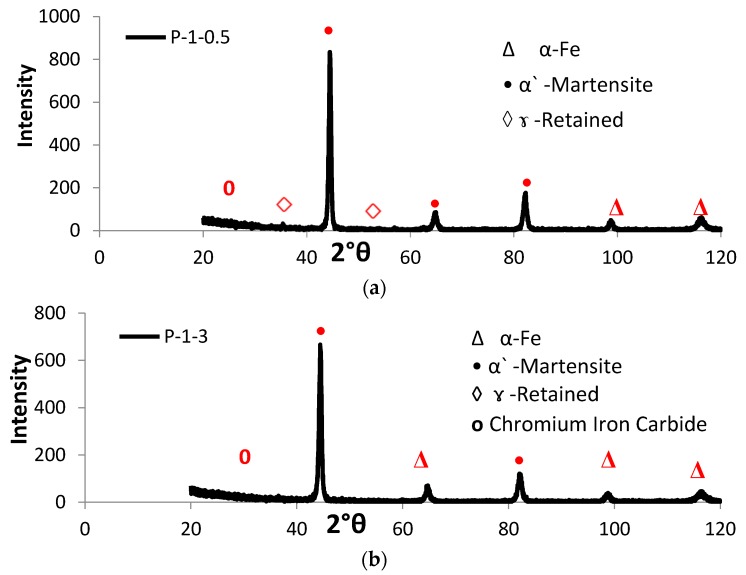
XRD spectra for various LHT AISI 416 specimens processed at laser power 1000 W and scanning speeds of (**a**) 0.5 and (**b**) 3 m·min^−1^.

Figure 5 shows typical optical micrographs of LHT samples treated at laser power of 1000 W. The surface layer of all specimens was free of cracks and pores. The EDX data of such samples were analyzed in Figure 6 for the condition indicated. The XRD spectra for the specimens under different processing conditions are shown in Figure 7. All LHT specimens were composed mainly of martensite, with retained austenite and small traces of the ferrite phase. Extra investigation via transmission electron microscopy (TEM) is planned for future work.

**Figure 8:**
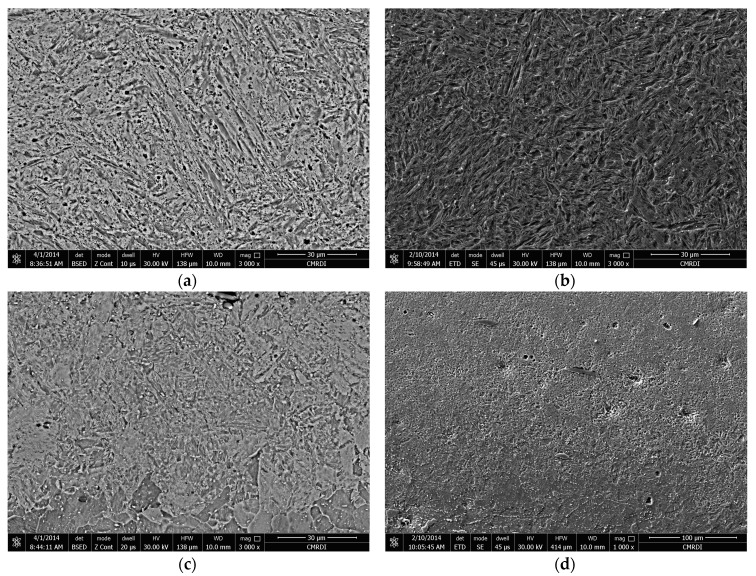
SEM microstructure (magnification 1000×): (**a**) HZ; (**b**) HAZ of sample P-07-0.5 and (**c**) HZ; (**d**) HAZ of sample P-1-0.5.

Additional information of both hardened zones (HZ) and heat affected zones (HAZ) was gained from the light micrographs of the laser surface treated specimens at a higher magnification using SEM, as presented in Figure 8.

**Figure 9:**
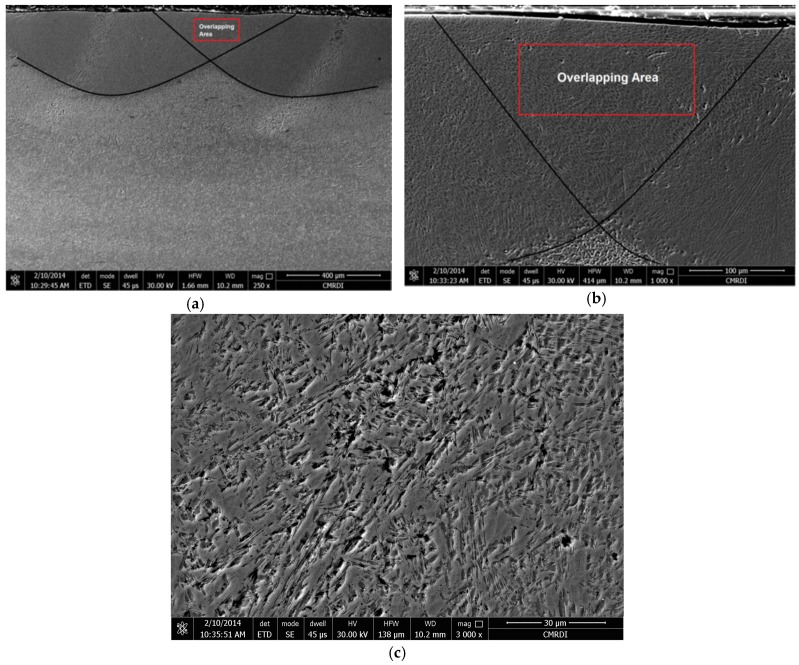
SEM micrographs of the highlighted overlapping area of sample P-1-2, at magnifications of (**a**) 250×; (**b**) 1000×; (**c**) 3000×.

As mentioned before, the optimal smoothness of track profiles was acquired by adopting an overlap of 25%. No defects were recorded, as seen in Figure 9. The obtained results coincided with previous reports, for example, [25]. The microstructure consisted of mixture of martensite and retained austenite.

### 3.2. Hardened Depth

**Figure 10:**
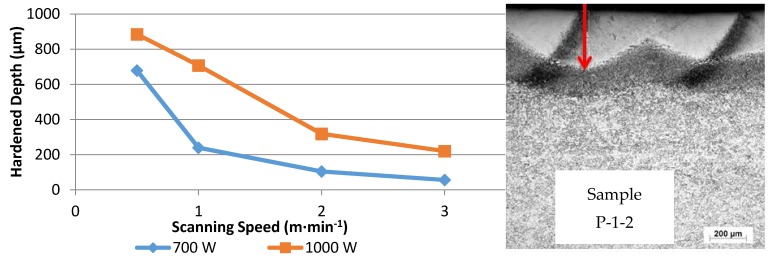
Variations in the depth of hardened regions with respect to laser power and laser scanning speed.

The hardened depths were evaluated under the optical microscope at magnification 50× from the highest point to the deepest point in the hardened zone, as shown in Figure 10. The hardened depths were measured at exactly the same location (in the middle of the hardened layer) in all samples. The hardened depth due to laser hardening was directly affected by the laser power and beam spot diameter (power density W·mm^−2^) [26]. As the power density increased and the transverse speed decreased the hardened depth and width increased.

### 3.3. Macrohardness 

The hardness value of the base material was in the range of 155–160 HV, and the hardness value of the CHT samples was in the range of 500–514 HV. Both cases showed uniformity of hardness distribution. On the other hand, laser treated samples exhibited variations in hardness distribution, being in the range of 440–510 HV. Heat input values were calculated to combine the effect of laser power with the scanning speed and the laser beam diameter into one value. Heat input was calculated from the following equation H = p/d.v where: H: heat input (J·mm^−2^), p: laser power (J·s^−1^), d: beam diameter (mm), and v: laser scanning speed (mm·s^−1^) [4].

**Figure 11:**
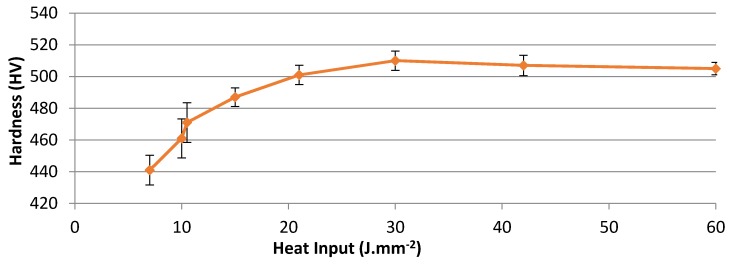
Heat input values versus average hardness values of laser surface treated AISI 416 samples. Error bars represent standard deviation.

It was indicated that at constant laser scanning speed, samples treated at higher laser powers (1000 W) had higher hardness values than samples treated at lower laser powers (700 W). The present experimental findings (as shown in Figure 11) showed that the obtained surface hardness strongly depended on the values of heat input.

### 3.4. Microhardness Distribution

**Figure 12:**
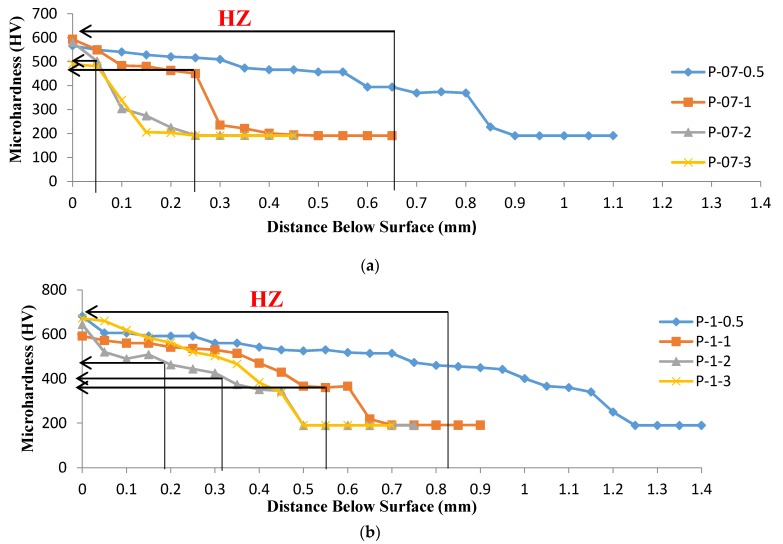
The microhardness profiles of the laser-hardened specimens treated at laser powers of: (**a**) 700 W; and (**b**) 1000 W, corresponding to different laser scanning speeds (0.5, 1, 2 and 3 m·min^−1^).

**Figure 13:**
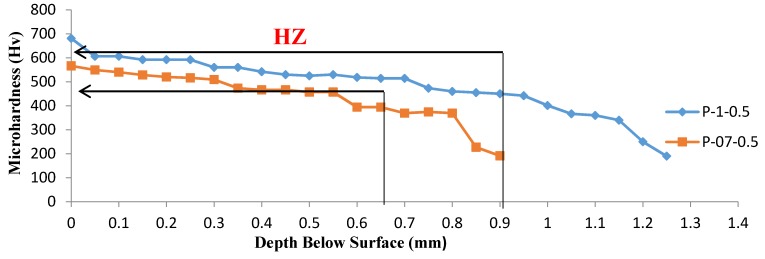
The microhardness profiles of laser treated samples processed at two different laser powers (700 and 1000 W) at a constant laser scanning speed of 0.5 m·min^−1^.

Figure 12 and Figure 13 display the Vickers microhardness depth profiles for laser hardened samples. All profiles could be divided into three zones corresponding to the microstructure, the hardened zone (highlighted in figures), the heat-affected zone, and the base metal zone, respectively. 

### 3.5. Wear Behavior

**Figure 14:**
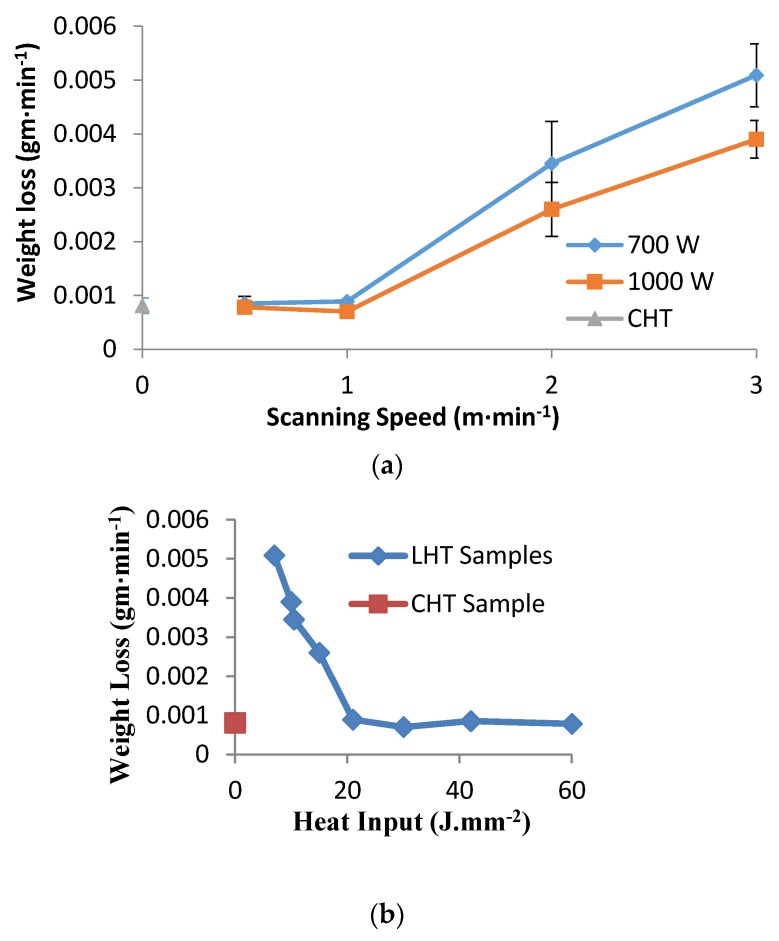
Wear behaviors of CHT and LHT samples, (**a**) weight loss vs. scanning speed; (**b**) weight loss vs. heat input.

The loss of weight as a result of wear is shown in Figure 14 with error bars representing standard deviation. In this figure, the wear resistance of the LHT samples was compared with those of the CHT samples. Up to a laser scanning speed of 1 m·min^−1^, the wear resistance of laser treated samples, at both powers, was comparable to conventionally hardened samples. On the other hand, LHT samples exhibited a decrease in wear resistance as laser scanning speed increased. It was demonstrated from the curve of the weight loss versus the heat input (Figure 14) that the weight loss decreased with an increase in heat input. This was reflected through the surface hardness values after the laser surface treatment (Figure 11).

### 3.6. Impact Results

**Figure 15:**
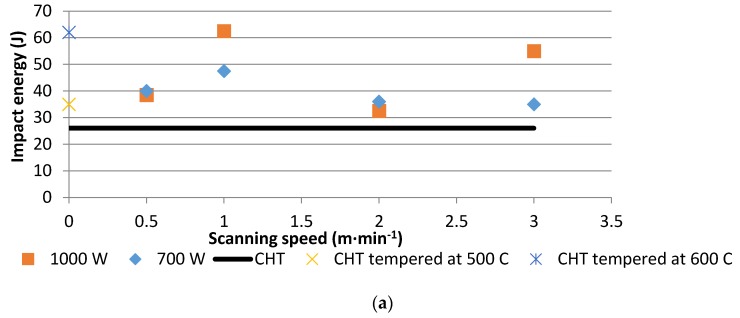

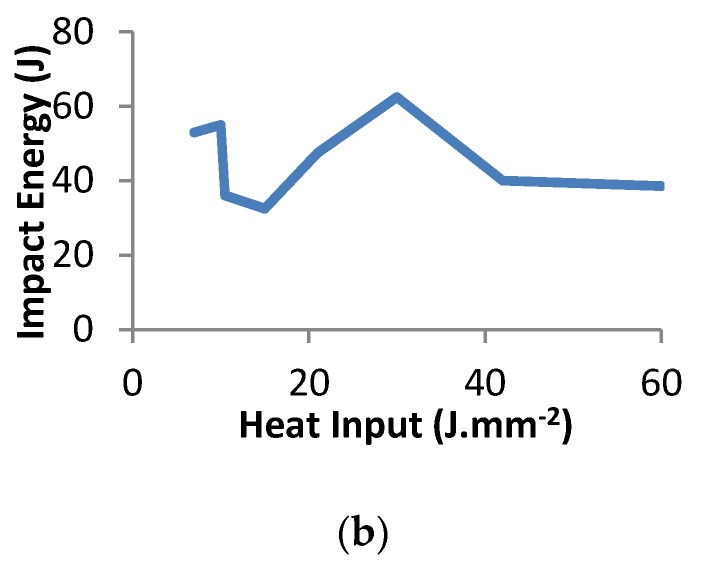
The impact energy profiles of CHT and LHT samples, (**a**) impact energy vs. laser powers and laser scanning speeds; (**b**) impact energy vs. heat input.

Figure 15 presents impact energy values for CHT and LHT samples at different laser hardening conditions. In this work, the tempering of the CHT samples at two different temperatures of 500 and 600 °C was performed in order to compare the impact energy of the LHT samples with the hardened plus tempered martensite stainless steel, as normally treated.

### 3.7. Corrosion Results

**Figure 16:**
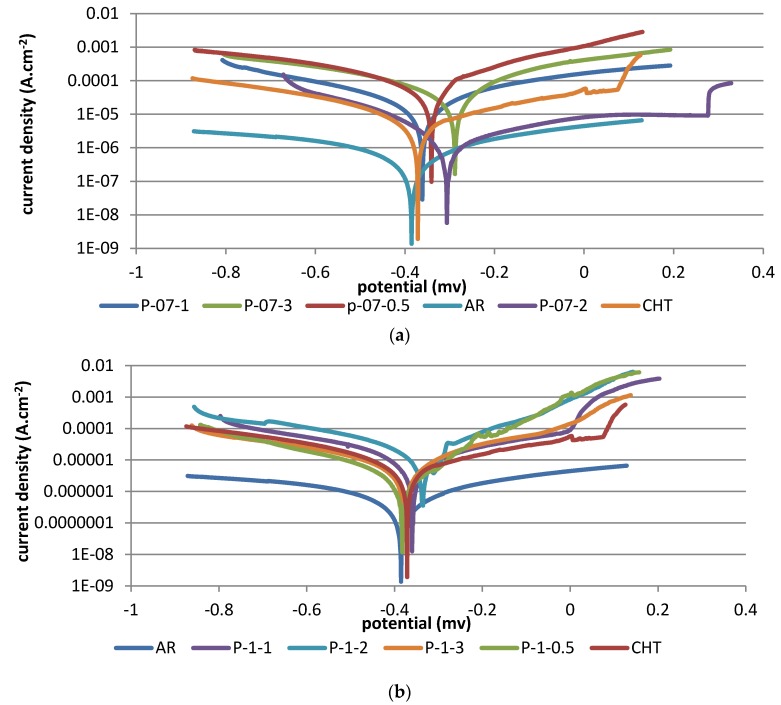
Potentiodynamic polarization curves of AR 416, CHT, and LHT samples treated at four different laser scanning speeds at laser powers of: (**a**) 700 W; and (**b**) 1000 W.

Figure 16a,b shows typical potentiodynamic polarization curves for AR, CHT, and LHT at laser powers of 700 W and 1000 W, respectively. The corrosion potentials obtained from these curves were presented in Table 2. 

## 4. Discussion

### 4.1. Microstructural Analysis

Laths of martensite were formed in the conventionally treated samples, due to the slower cooling rate from austenite compared to laser surface treatment, as discussed below. As for the laser treated samples, the microstructures observed in this work were in accord with those mentioned previously [21]. The morphology of the cross section of the laser affected region could be divided into three regions based on their different microstructures: (i) the hardened zone, (ii) the heat affected zone and (iii) the base metal, respectively. 

SEM showed that the HZ consisted of martensite containing a small amount of retained austenite (identified with the help of microhardness measurement). The HAZ consisted of martensite, carbide particles, MnS inclusions, and traces of initial ferrite grain structures. The results were in good agreement with those reported from XRD analysis.

The principal observation from the EDX data of the HZ, HAZ and BM was that there were higher sulfur and manganese contents in the HAZ as compared to the HZ and BM; this could be attributed to the high laser power and slow scanning speed used, because in this condition, partial melting may have occurred and resulted in diffusion of sulfur and manganese from the hardened zone toward the heat affected zone. 

The present data recorded a shallower hardened zone accompanying the high laser scanning speeds, and the opposite occurred at slower scan speeds. This was understood as a high laser scanning speed leading to less energy being applied to the substrate. 

### 4.2. Hardness and Wear Behavior

The variations in heat input values may have been the reason for the differences in the hardness values [27]. The hardness values increased with increasing heat input, or decreased with increasing laser scanning speed, as shown in Figure 11. Changing the surface absorption coefficient of the laser may cause differences in absorbed laser beam energy values, especially in the case of multi-pass heating with overlapping tracks [3]. Selective hardening advantageously traps the diffused free carbon in the steel via both rapid heating and quenching; this makes the steel harder than in normal phase transformations [21].

At high heat input above 21 J·mm^−2^, there was sufficient heat to obtain a complete austenitization process before quenching, which meant that the carbon dissolved completely in the austenite. Therefore, the martensite thus formed exhibited higher hardness values. On the other hand, at heat inputs lower than 21 J·mm^−2^, carbon dissolution in austenite was incomplete before cooling occurred, and this austenite transformed into martensite with lower hardness values. Also it could be seen that, for heat input <21 J·mm^−2^, the energy absorbed by the surface was not large enough to heat the material surface to the required quenching temperature (above 960 °C) [28], as had been noted by Tianmin et al. [16]. Therefore, hardness values decreased below 500 HV.

The microstructure of the hardened zone consisted mainly of martensite with microhardness values of approximately HV 700. The heat affected zone, which contained primary ferrite structures, exhibits microhardness values of approximately HV 450, which was higher than that of HV 200 in the AISI 416 martensitic stainless steel alloy substrate, as a result of martensitic transformation which occurs during laser treatment. Microhardness decreased significantly from the hardened surface to the substrate. A difference in the resulting hardened depth at constant laser scanning speed and different powers was observed, from the data presented in Figure 12 and Figure 13. By taking a cutoff at HV 500, it was evident that depth values of 0.7 mm and 0.3 mm were obtained at a laser scanning speed of 50 cm·min^−1^ and powers of 1000 and 700 W, respectively. 

The variation of surface hardness with heat input reported in Figure 11 supported the wear behavior of the specimens under different laser parameters. When the hardness level increases, the wear resistance also increases [29,30]. Furthermore, as seen in Figure 12 and Figure 13, the depth of hardening was reduced at lower laser powers and at higher laser scanning speeds (lower heat input conditions). The more weight loss observed in Figure 14, which was associated with lower laser power and higher laser scanning speeds, may have been also partially related to shallower hardened depth, which was likely to be entirely worn–out during the wear test. The weight loss measured in this case could include both the hardened zone as well as the tougher heat affected zone (HAZ) which contained some untransformed ferrite. This might explain the increasing weight loss with the decreasing heat input of the processing laser.

It is noteworthy that at scanning speed 1 m·min^−1^ for both laser powers, the corresponding samples showed comparable weight losses, in spite of the considerable difference of hardened depth in both cases (700 vs. 240 µm for 1000 and 700 W, respectively). This anomaly may suggest that the worn surface layer was less than 240 µm in both cases, and therefore the effect of laser power appeared insignificant.

Based on the mentioned experimental results (Figure 11 and Figure 14), the wear resistance not only depended on the hardened depth, but also on other factors, such as the amount of heat input and the hardness level. Additionally, compressive stresses generated from laser hardening also add to factors controlling abrasive wear resistance [3]. The high stresses induced during rapid cooling of the surface hardened layer tended to create fine and highly dislocated martensite, with some level of stable austenite.

### 4.3. Impact Toughness

The measured impact energy for laser treated samples actually represented the overall effects contributed by both the hardened zones and the tough interior base metal (structure somewhat similar to a composite material [15]), unlike conventionally heat treated samples. The same level of wear resistance was achieved in both conditions. Clearly the impact energy tended to increase between the heat input range of 15–30 J·mm^−2^ (Figure 15). This observation was in accord with previously reported results although that the laser type was a Q-switched pulsed laser, in contrast with the present continuous laser [31]. The reason for this increase was due to the residual compressive stress induced by a laser surface treatment which was able to restrain the growth of the crack. However, the decrease of the impact energy for a heat input larger than 30 J·mm^−2^ needs more study to be sure that it is a recurring phenomenon. 

The improved toughness of the laser-hardened specimens was also attributed to the refined microstructures with lower MnS and carbide particles concentrations along the prior austenite grain boundaries in the laser-hardened zone (as identified by EDX, Figure 6) [15].

The present data also showed that CHT samples tempered at 500 °C and 600 °C had impact energies of 35 and 62 J, respectively. The reason for these different values was that during tempering at 500 °C, a secondary hardening may have occurred in the stainless steel, due to carbide precipitations [32]. CHT samples tempered at 600 °C had comparable impact energy with LHT samples, but had much lower hardness levels (280 Hv) than the hardness levels achieved in LHT samples (510 Hv). A remarkable achievement in the impact energy (more than twice) could be noticed at a laser scanning speed of 1 m·min^−1^ and a laser power of 1000 W, as compared to CHT conditions (after tempering at 500 °C). Lower wear resistance is expected to accompany the lower hardness of the tempered CHT specimens. 

It should be pointed out that the data presented here concerning impact was rather limited, and mainly directed towards comparing the impact toughness levels of the conventional and the laser treated samples. However, we conclude drawn that the results have some practical significance for impact toughness, as our objective was to find the heat input values that would lead to optimum mechanical and physical properties (hardness, wear resistance, impact toughness, and corrosion resistance).

### 4.4. Corrosion Resistance

The corrosion performance of AISI 416 martensitic stainless steel is essentially determined by two factors: (a) the amount and size of carbide particles and manganese sulfide inclusions on the grain boundaries of the hardened zone; and (b) the refinement of microstructure [20]. Chromium carbides are rich in this type of steel, because chromium is a dominant carbide-forming element. The higher the carbide level, the more the chromium is bounded in carbides, which results in less chromium in the solid solution for the formation of the passive film. Furthermore, the corrosion resistance increases with an increasing degree of microstructure refinement. 

It could be observed from Figure 16 that corrosion potentials for all samples were very similar, and showed that LHT treatment did not change the fundamental nature of the passive film. Therefore from this standpoint, LHT of AISI 416 is capable of enhancing corrosion resistance by reducing the amount and size of manganese sulfide inclusions and carbide particles in the hardened zone, as compared to the CHT samples. Data in Table 2 shows that LHT improved the corrosion potentials of grade AISI 416 stainless steel by 80 and 70 mV over that verified for the AR and CHT samples, respectively. These results agreed with the findings reached by Yong [21]. The corrosion potential of some LHT samples shifted towards an optimum direction. According to the polarization curves in Figure 16, it could be supposed that the optimum condition which provided the best corrosion resistance was P-07-200 at a heat input of 10.5 J·mm^−2^, and this could be attributed to a matrix having a microstructure consisting of the appropriate proportions of martensite, finer carbides, retained austenite, and lower sulfur and different oxides. Such a microstructure displayed a promising combination of toughness and strength, and contained less weak sites for corrosion attack.

## 5. Conclusions

1. Laser surface treatment of AISI 416 martensitic stainless steel considerably increased the surface hardness from 155 to 510 HV. The obtained hardness values were found to strongly depend on the heat input values. The hardness values increased with increasing heat input, or decreased with the increasing laser scanning speed.
2. The microhardness distribution in the laser affected region showed a stepwise profile. The average microhardness in the hardened zone reached HV 700, and in the heat affected zone it reached HV 450. The size of the shallowest hardened layer was 57 μm, and was associated with the lowest heat input (7 J·mm^−2^), while the deepest hardened layer was 885 μm and was achieved with the highest heat input value (60 J·mm^−2^), both with the lowest laser scanning speed of 0.5 m·min^−1^.
3. The laser affected region could be divided into two zones based on different microstructures: (i) the hardened zone and (ii) the heat affected zone.
4. As for heat input above 21 J·mm^−2^, heat was sufficient to obtain complete austenitization, and the carbon completely dissolved in austenite and caused an increase in hardness values and vice versa.
5. Wear results showed that up to scanning speed of 1 m·min^−1^, the wear resistance of samples processed at both laser powers was comparable to the conventionally hardened samples. On the other hand, at higher speeds, LHT samples showed a decrease in wear resistance as the scanning speed increased; this may have partially been related to a shallower hardened depth, which might have entirely worn out during the wear test. The weight loss decreased with the increase in heat input. This was reflected through the surface hardness values after laser surface treatment.
6. The wear resistance depended on the hardened depth, the amount of heat input, hardness levels, and compressive stresses.
7. A significant result concerning the impact toughness and hardness values of laser heat treated samples, as compared to conventional heat treated samples, was reported. At the same level of hardness, laser treated samples experienced more than double the improvement in impact toughness. The reason for this increase was due to residual compressive stress induced by a laser surface treatment, which was able to restrain the growth of cracks.
8. The CHT samples tempered at 600 °C had comparable impact energy with LHT samples, but at much lower hardness levels (280 Hv vs. 510 Hv), with correspondingly higher wear resistance in LHT samples.
9. The corrosion potentials of the LHT samples improved by 80 and 70 mV over those recorded for the AR and CHT samples respectively.
10. The optimum condition for wear resistance, impact toughness and corrosion resistance was recorded at a heat input value of 21 J·mm^−2^. This may not have been the maximum value for each property, but it was the general optimum, consideringall of the properties as a whole.

This paper threw light on the significance of laser treated samples as compared to conventional treated samples, particularly in regards to impact toughness, wear resistance, and corrosion resistance, under the conditions specified.

## Figures and Tables

**Table 1:** The laser surface treatment parameters.

Sample No. P-X-Y	Laser Power P (KW)	Laser Scanning Speed v (m·min^−1^)
P-07-0.5	0.7	0.5
P-07-1	0.7	1
P-07-2	0.7	2
P-07-3	0.7	3
P-1-0.5	1	0.5
P-1-1	1	1
P-1-2	1	2
P-1-3	1	3

**Table 2:** Corrosion potentials determined from potentiodynamic polarization curves.

Condition	AR	CHT	P-07-0.5	P-07-1	P-07-2	P-07-3	P-1-0.5	P-1-1	P-1-2	P-1-3
Corrosion potential (mV vs. SRE)	−385	−372	−340	−359	−307	−328	−380	−361	−335	−378
